# Ultrasonic-Assisted Extraction of Raspberry Seed Oil and Evaluation of Its Physicochemical Properties, Fatty Acid Compositions and Antioxidant Activities

**DOI:** 10.1371/journal.pone.0153457

**Published:** 2016-04-27

**Authors:** Hui Teng, Lei Chen, Qun Huang, Jinli Wang, Qiyang Lin, Mingxin Liu, Won Young Lee, Hongbo Song

**Affiliations:** 1 College of Food Science, Fujian Agriculture and Forestry University, Fuzhou 350002, China; 2 Food and Bio-Industry Research Institute, Kyungpook National University, Daegu 702–701, Republic of Korea; 3 School of Food Science and Bio-Technology, Kyungpook National University, 1370-Sankyunk Dong, Puk Gu, Daegu 702–701, Korea; College of Agricultural Sciences, UNITED STATES

## Abstract

Ultrasonic-assisted extraction was employed for highly efficient separation of aroma oil from raspberry seeds. A central composite design with two variables and five levels was employed and effects of process variables of sonication time and extraction temperature on oil recovery and quality were investigated. Optimal conditions predicted by response surface methodology were sonication time of 37 min and extraction temperature of 54°C. Specifically, ultrasonic-assisted extraction (UAE) was able to provide a higher content of beneficial unsaturated fatty acids, whereas conventional Soxhlet extraction (SE) resulted in a higher amount of saturated fatty acids. Moreover, raspberry seed oil contained abundant amounts of edible linoleic acid and linolenic acid, which suggest raspberry seeds could be valuable edible sources of natural γ-linolenic acid products. In comparison with SE, UAE exerted higher free radical scavenging capacities. In addition, UAE significantly blocked H_2_O_2_-induced intracellular reactive oxygen species (ROS) generation.

## Introduction

Raspberry (Rubus coreanus Miquel), referred as bokbunja in Korea, belongs to the family of Rosaceae and is distributed only in southeast Asian countries, especially in southern parts of the Korean peninsula, China, and Japan[1,2]. Raspberry fruits are popularly consumed not only in fresh and frozen forms but also as processed and derived products, including dried and canned fruits, yogurts, beverages, jams, jellies, and wines [3,4]. Raspberry seeds are an important byproduct in the production process of raspberry wines and juices, but usually be discarded and thus underexploited. Various researchers have discovered that raspberry seed oil may be a promising biomaterial with various beneficial properties. Johansson et al. [5] reported that raspberry seeds constitute 9–12% of the weight of raspberry fruits, and a high oil recovery rate of 10–23% from raspberry seeds was obtained. A previous study [6] also confirmed that raspberry seed oil contains various functional compounds such as phytosterol, linoleic, α-linoleic, oleic, and palmitic acids. Additionally, it has been found that raspberry seed oil contains superior levels of tocols, leading to higher anti-inflammatory and antioxidant activities as compared to other oil products such as grape seed, safflower, wheat germ, and avocado oils [7,8]. Therefore, extraction of essential raspberry seed oil from these waste materials could help solve environmental problems and expand the raspberry market.

Until now, many extraction techniques have been utilized for the extraction of essential oils from different plant seeds. Conventional methods such as Soxhlet extraction and cold-press extraction have been used [9–11]. However, these methods are very time-consuming and require large quantities of solvents [12,13]. More advanced extraction methods have been developed and adopted. Ultrasonic-assisted extraction (UAE) is a rapid and effective extraction technique that uses ultrasound to generate rapid movement of solvents, resulting in a higher mass transfer speed as well as acceleration of extraction [14]. Compared to other advanced extraction techniques such as supercritical fluid extraction and ion-pair extraction, UAE is more economic, eco-friendly, and convenient. And recently, there have been many reports on the application of UAE in extraction of trace organic compounds from soil, animal and plant tissues. Our previous study [15] employed UAE for anthocyanin and polyphenol extractions from raspberry flesh suggesting that high extraction yield with condensed extraction time. UAE has also been successfully used for functional compounds extraction from kindred berry fruits such as black chokeberry and haskap berry [16,17]. Nevertheless, several process variables such as extraction temperature, processing time, medium solvent, and solvent-to-sample ratio are usually considered as important factors when optimizing extraction process. As such, different combinations of process variables could have significant effects on the extraction yields of functional compounds from the raw materials.

To solve this problem, a statistical method known as response surface methodology (RSM) was applied to determine the best combination of process parameters to ensure maximal extraction efficiencies. This method is commonly used to establish the experimental conditions of process parameters based on a second order polynomial model. According to the fitted polynomial model, a response surface plot was generated to determine the optimal conditions and maximal extraction yields. Compared to other statistical methods such as orthogonal design method and single factor experiment method, RSM can reduce the number of experimental trials and determine the interactive effects of process variables [18].

Thus, the objective of this study was to employ highly efficient UAE of raspberry seed oil. The effects of process variables such as extraction temperature, solvent types, and sonication time on oil yield, vitamin E, and antioxidants from raspberry seeds were investigated. Significant extraction variables were determined, and optimal conditions of UAE were predicted by using RSM.

## Material and Methods

### Raspberry seed and flesh separation

Raspberry fruits (*Rubus coreanus* Miq.) freshly harvested from Mungyeong (latitude: 128°2’, longitude: 36°6’) in the Gyeongbuk area of Korea were purchased. Fruit sample were harvested from May to June 2014 at fully maturity, the color of raspberry fruits were blood red with sphere appearance, but hollow inside. The raspberry sample was lyophilized using a freeze dryer (FD-5508, Ilshin Lab Co, Seoul, Korea). The freeze-dried sample was hand-crushed and passed through a 25-mesh sieve in order to separate seeds from raspberry flesh. Then, lyophilized raspberry flesh was grounded into powder using a grinder, passed through a 40-mesh sieve, packed in a polyethylene zipper bag, and kept at -18°C. Raspberry seeds were then soaked with cold tap water for approximately 24 h. After soaking, residual berry pulp was manually separated gently from the seeds. Then, pigments were washed off from the seeds using tap water until the washing water was clear, after which the moisture was drained off in a convection oven at 60°C for 24 h. Then, dried raspberry seeds were milled into powder, passed through 40 mesh screen, collected in a zipper bag, and stored in a refrigerator. Moisture content of raspberry seeds was determined using a drying oven set at 105°C.

### Soxhlet extraction (SE) process

Ten grams of raspberry seed flour were added into a cellulose thimble (28 mm i.d. * 100 mm long, Advantec, Tokyo, Japan) and placed into a Soxhlet apparatus that was connected to a 250 mL round bottom flask containing 200 mL organic solvent. The extraction process lasted for 4 hrs until the solvent in the reflux pipe became colorless. The extract was then evaporated under vacuum and dehydrated with anhydrous sodium sulfate, after which the collected essential oil was stored in an amber-colored vial under nitrogen at -20°C until further analysis.

### Ultrasonic-assisted extraction (UAE) process

A sonication cleaning bath (JAC Ultrasonic 2010P, Jinwoo Engineering Co., Ltd., Hwasung, Gyeonggi, Korea) was used for UAE. The device was operated at a frequency of 40 kHz, an ultrasonic input power of 250 W, and a useable volume of 10 L (internal dimensions: 300×240×150 mm). The available range for heating was from 0 to 70°C. Briefly, 1.0 g of milled raspberry seed powder was placed in a 100-mL Erlenmeyer flask and mixed with 40 mL of extraction solvent, after which the flask was placed in the sonication bath for extraction. Upon completion of extraction, the extract was removed from the vessel and filtered using No.1 filter paper under vacuum conditions. The raspberry seed filtrate was dehydrated using anhydrous sodium sulfate and then diluted to 50 mL using the same organic solvent used in the extraction process. Extraction yield of raspberry seed oil was expressed as % of dry weight of raspberry seeds.

### Extraction yield

Extraction yield of raspberry seed oil was analyzed using the gravity method described by the AOAC official method 922.10 [19]. A 5 mL aliquot of raspberry seed extract was transferred into an aluminum plate, which was dried at 105°C until all moisture was removed.

### Vitamin E determination

Vitamin E analysis was carried out according to the spectrophotometric method described by Prieto et al. [20] based on the formation of a green-colored complex of phosphate and Mo (V). Raspberry seed oil (100 μL) was properly diluted with hexane and placed in a glass tube, after which 1 mL of reaction reagent consisting of 0.6 M sulfuric acid, 28 mM sodium phosphate, and 4 mM ammonium molybdate was added. After incubation at 37°C for 90 min, the absorbance was read at 695 nm. *α*-Tocopherol was used as a standard, and the results were expressed as mg/g dw (dry weight).

### Fatty acid analysis

#### Methylation procedure

Fatty acid methyl esters (FAME) were prepared by following the IUPAC methodology without heating. Raspberry seed extract (50 mg) was accurately weighed in a 10-mL volumetric flask and then dissolved in a mixed solvent of ethyl ether and benzene (1:1). A 2 mL aliquot of 0.4 mol/L KOH solution (made in methanol) was added to the solution, which was mixed vigorously and kept still at room temperature for 30 min. Finally, saturated NaCl solution was added until FAME floated to the neck of the volumetric flask. Then, a 1 mL aliquot of supernatant was collected and filtered through a 0.45-μm nylon membrane for gas chromatography (GC) analysis.

#### GC-MS analysis for fatty acid

GC analysis was conducted according to the method described by Parry et al. [21]. A fused silica HP-5 column (30 m×0.25 mm id, 0.25 μm, Hewlett Packard) was used, and hydrogen was used as the carrier gas at a flow rate of 1.0 mL/min. Oven temperature was maintained at 75°C for 2 min, increased at 5°C/min to 175°C, held for 33 min, increased at 5°C/min to 225°C, and then held for 15 min. The mass spectrum (MS) was operated under 70 eV with a scan range of 15–500 amu. Chemical composition of raspberry seed extract was identified by matching the mass spectra with the data in the NIST02 Mass Spectral Library.

### Organic radical scavenging activities

#### DPPH radical

The antioxidant effects of raspberry seed oil were analyzed by using DPPH assay [22]. Raspberry seed oil (100 μL) was mixed with 900 μL of pure DPPH (100 μM) solution dissolved in pure ethanol. The sample was then shaken vigorously and kept in the dark at room temperature for 30 min. Control was prepared using distilled water instead of sample. The absorbance was measured at 517 nm, and the results were expressed as mmol/trolox equivalent/g dry weight (mmol TE/g dw).

#### ABTS radical

The total antioxidant activity was measured by the ABTS^+^ radical cation decolourisation assay (Chen & Kang, 2014). Distilled water was used to dissolve ABTS to 7 mM concentration, and the ABTS radical cation (ABTS^+^) produced by reacting with 2.45 mM potassium persulfate and the mixture was left in darkness at room temperature. After 12 h, the absorbance was adjusted to 0.70 ± 0.05 at 734 nm by adding absolute ethanol. To determine the scavenging activity, 190 μL of ABTS reagent was mixed with 10 μL of extracts, and absorbance was measured after 6 min of reaction at room temperature, using trolox as a control.

#### NO radical

NO generated from sodium nitro ferricyanide dihydrate (SNP) was measured by the Griess reagent. As described by our previous study (Chen & Kang, 2014), ach 25 μL sample or quercetin was mixed with 25 μL of 10 mM SNP in PBS (pH 7.4), incubated at 25°C for 150 min, and then added 50 μL of Gress reagent (2% sulfanilamide in distilled water and 0.2% naphthyletylene-diamine dihydrochloride in 4% phosphoric acid). DMSO was used instead of test samples as a control. The absorbance of the chromophore formed during the diazotization of nitrite with sulphanilamide and subsequent coupling with naphthylethylene-diamine was read at 540 nm, and referred to the absorbance of standard solutions of sodium nitrite treated in the same way with Griess reagent. The plot between the concentration of nitrite and incubation time exhibited the best incubation time for nitrite production from SNP.

### Cell culture and cellular reactive oxygen determination

The RAW 264.7 cell line was obtained from Korea Cell Line Bank (Korea). Cells were maintained in Dulbecco’s Modified Eagle’s Medium (DMEM) supplemented with 10% FBS, 100 units of penicillin, and 100 streptomycin sulfates at 37°C in an atmosphere of 5% CO_2_. Cellular reactive oxygen was determined as previous study (Cheng et al., 2015) with minor modification. Intracellular ROS level was assessed using DCFH-DA as fluorescent label. RAW 264.7 macrophages were seeded at a density of 2 × 10^5^ cells per well in a 96-well culture plate. The cells were treated with H_2_O_2_ after being pretreated with or without tested samples for 12 h. After incubation, the cells were washed with phosphate buffered saline (PBS, pH 7.4). DCFH-DA (final concentration 50 μM) diluted in DMEM without phenol red was added to the cells and then incubated for 60 min at 37°C. After incubation, the cells were washed with cold-PBS and the fluorescence intensities of the stained cells were determined in a absorbance at 485 and 535 nm (excitation and emission wavelengths, respectively) was measured using Fluorescence spectrophotometer SpectraMax M5 (Molecular Devices, CA, USA) and using flow cytometry using CellQuest Pro Acquisition software (Becton Dickinson; Franklin Lanes, NJ, USA) equipped the FACSCalibur system^TM^ (BD Biosciences, San Diego, USA) analyzed

### Experimental design

Central composite design was used to arrange the experiments and optimize the extraction of raspberry seed oil subjected to UAE. Under most circumstances, only pure organic solvents are considered for essential oil extraction from plant seeds due to the lipophilic properties of lipids and fats, and using pure organic solvent can avoid unnecessary contamination of essential oils. Peres et al. [13] confirmed that ethanol results in higher extraction yields of vitamin E, linoleic acid, and α-linolenic acid, which are the major compounds in raspberry seeds, from medical plants. Therefore, ethanol was used for UAE of raspberry seed oil, and extraction temperature (30–70°C) and sonication time (10–50 min) were the two variables. Regression analysis was performed on data obtained from triplicate measurements of dependent variables. Response surface analysis was applied to data obtained from CCD matrices in order to calculate and determine the optimum conditions for UAE of functional compounds from raspberry flesh and seeds.

The process variables Xi were coded as xi according to the equation below:

$$x_{i}=\left(X_{i}-{\bar{X}}_{i}\right)/\Delta X_{i}$$ (1)

Where *x*_*i*_ is the coded level, *X*_*i*_ is the natural level of the independent variables, ${\bar{X}}_{\text{i}}$ is the mean of the natural level of the independent variables, and ΔX_i_ is the step change value.

Experimental data were fitted into an empirical second order polynomial model using regression analysis and presented in the following equation:

$$Y=\beta_{0}+\sum_{i=1}^{k}\beta_{i}x_{i}+\sum_{i=1}^{k}\beta_{ii}x_{i}^{2}+\sum_{1\leq i\leq j}^{k}\beta_{ij}x_{i}x_{j}+\varepsilon$$ (2)

Where *Y* represents the independent responses, *ß*_*0*_, *ß*_*i*_, *ß*_*ii*_, and *ß*_*ij*_ represent the regression coefficients of the process variables for the intercept, linear, quadratic, and cross product terms, respectively, and *ε* represents error. Statistical significance of the coefficients in the regression equation was checked by analysis of variance (ANOVA). The fitness of the polynomial model equation to the responses was evaluated by the coefficient of R-squared along with the F-test.

### Morphological observation of raspberry tissue

To better understand the extraction mechanism of action, morphological differences among raspberry seed tissues obtained by various extraction methods were investigated using scanning electron microscopy (SEM, S-4300, Hitachi, Japan). Raspberry seed samples extracted under optimal conditions for UAE and SE were collected and dried. After that sample were fixed on adhesive carbon tapes and then sputtered with a 4–5 nm gold-palladium layer. All sample were examined by scanning electron microscope under high vacuum conditions at an accelerating voltage of 15 kV.

### Statistical analysis

Analysis was carried out in triplicate and expressed as the mean ±SD (standard deviations). Significant differences as indicated were calculated using Student’s tests (*p*<0.05), and two-way analysis of variance (ANOVA) test (*p*<0.05 or *p*<0.001). All statistical analysis were prepared using SAS software (Version 9.2, SAS Institute Inc, Cary, NC), and all the Figs were generated using statistical software ((StatSoft, Inc., Tulsa, OK, USA).

## Results and Discussion

### Preliminary experimental for solvent choosing

**Fig 1:**
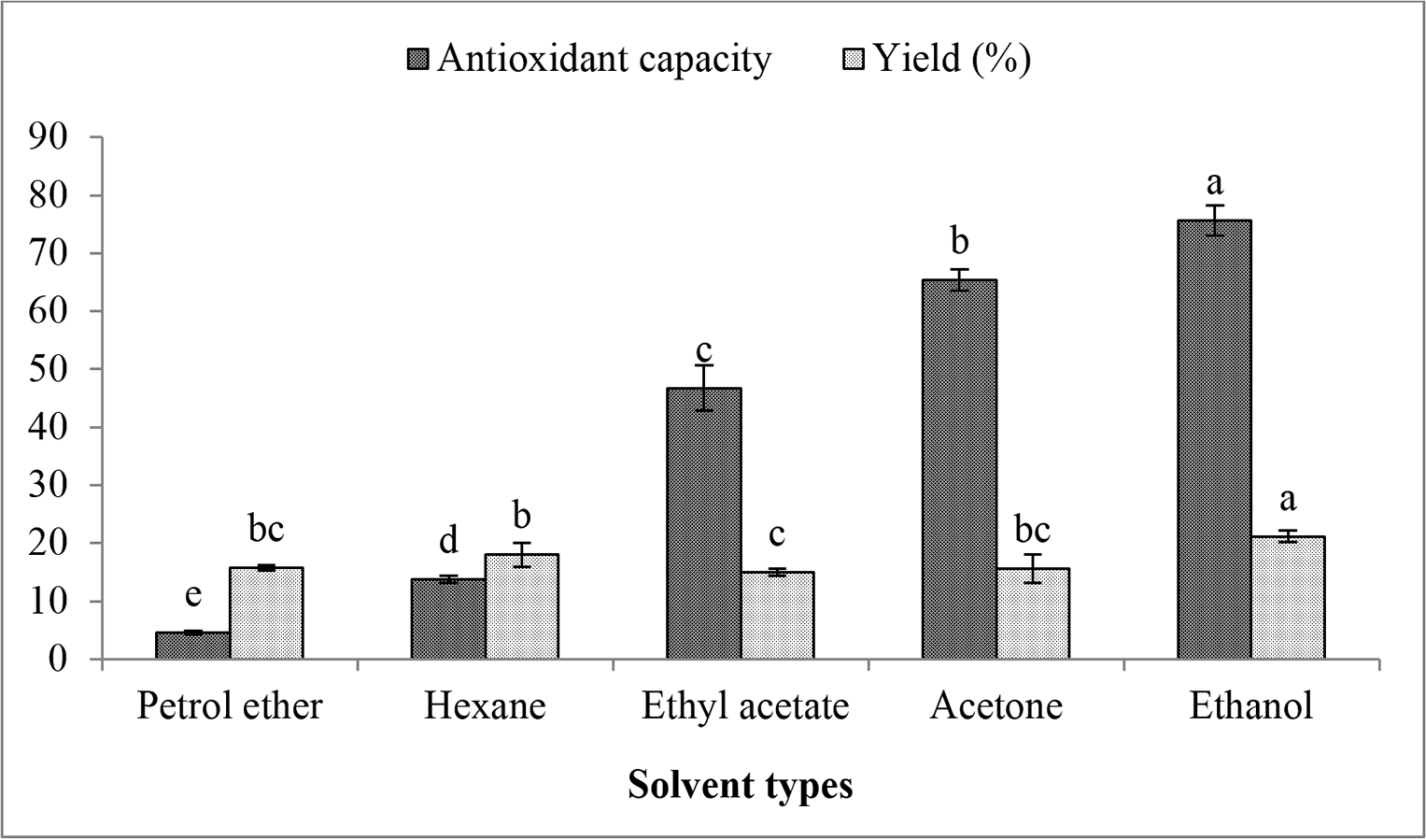
Effect of different solvent types on raspberry seed oil extraction under UAE.

According to relative study, UAE process involves 3 important variables including solvent types and concentration, sonication time and extraction temperature, which may significantly affect extraction efficiency. As for essential oil extraction, pure organic solvent alike petrol ether, hexane, ethyl acetate, acetone and ethanol are preferred due to less toxicity and no impurity compromising essential oil quality, thus preliminary experiments using different these 5 organic solvents were carried out. Fig 1 shows that ethanol extracted raspberry seed oil exhibited the highest antioxidant capacity and yield, followed by acetone and ethyl acetate, but petrol ether and hexane shows a weak ability for raspberry seed oil extraction under UAE. Thus, ethanol was selected as the appropriate extraction medium, and further UAE optimization study for raspberry seed oil focused on 2 important process variables of sonication time and extraction temperature.

### Model fitting

Sonication time and extraction temperature were considered as the main significant variables in the UAE process for raspberry seed oil extraction. The central composite design (CCD) based on two variables and five levels were generated for optimization. According to our preliminary test, the appropriate sonication time was between 10–50 min, and the UAE device had a temperature limitation of 70°C. Thus, an extraction temperature range of 30–70°C was used. CCD matrices and results for extraction yield, vitamin E content, and antioxidant activity are summarized in Table 1.

**Table 1:** Central composite design (CCD) for natural variable levels as well as experimental values of dependent responses of extraction yield and antioxidant capacity for raspberry seed oil using ultrasonic-assisted extraction.

Test.	Independent variable		Dependent responses		
	Sonication time (min)	Extraction temperature(°C)	Extraction yield ^a^ (%)	Vitamin E content ^b^(mg/g dw)	Antioxidant capacity ^c^(μmol TE/g dw)
1	20 (-1)	40 (-1)	19.70±0.56	13.11±0.15	60.11±1.04
2	20 (-1)	60 (+1)	22.10±0.33	14.16±0.08	72.02±2.12
3	40 (+1)	40 (-1)	22.15±0.18	14.43±0.11	73.49±1.44
4	40 (+1)	60 (+1)	22.79±0.24	14.67±0.17	77.90±1.54
5	10 (-2)	50 (0)	17.77±0.29	13.12±0.21	61.99±1.31
6	50 (+2)	50 (0)	22.16±0.11	14.60±0.17	75.02±0.52
7	30 (0)	30 (-2)	22.06±0.04	13.91±0.16	62.55±1.57
8	30 (0)	70 (+2)	22.86±0.22	14.08±0.09	65.45±1.36
9	30 (0)	50 (0)	23.01±0.21	15.00±0.05	79.30±1.53
10	30 (0)	50 (0)	22.59±0.19	14.97±0.16	79.90±1.31
11	30 (0)	50 (0)	21.92±0.15	15.10±0.18	79.90±1.48
12	30 (0)	50 (0)	22.59±0.14	14.81±0.03	85.38±1.38
13	30 (0)	50 (0)	22.83±0.16	14.79±0.07	82.04±1.85

^a^: coefficient of R square was 0.93 and lack of fit was 0.21 for fitted model of extraction yield

^b^: coefficient of R square was 0.89 and lack of fit was 0.14 for fitted model of vitamin E content

^c^: coefficient of R square was 0.90 and lack of fit was 0.14 for fitted model of antioxidant capacity

Regression coefficients for the fitted models of extraction yield, vitamin E content, and antioxidant capacity along with the ANOVA results are calculated. The fitted model for extraction yield showed the highest significance of 0.0007, and vitamin E content and antioxidant capacity also showed high significances of 0.0035 and 0.0022, respectively. R squares for extraction yield, vitamin E content, and antioxidant capacity were 0.93, 0.89, and 0.90, respectively. In addition, lack of fit for all three responses were insignificant with values greater than 0.05 (Table 1). These results suggest that the fitted models for all responses involved in UAE of raspberry seeds are reliable.

### Effects of process variables on extraction yield of raspberry seed oil

**Fig 2:**
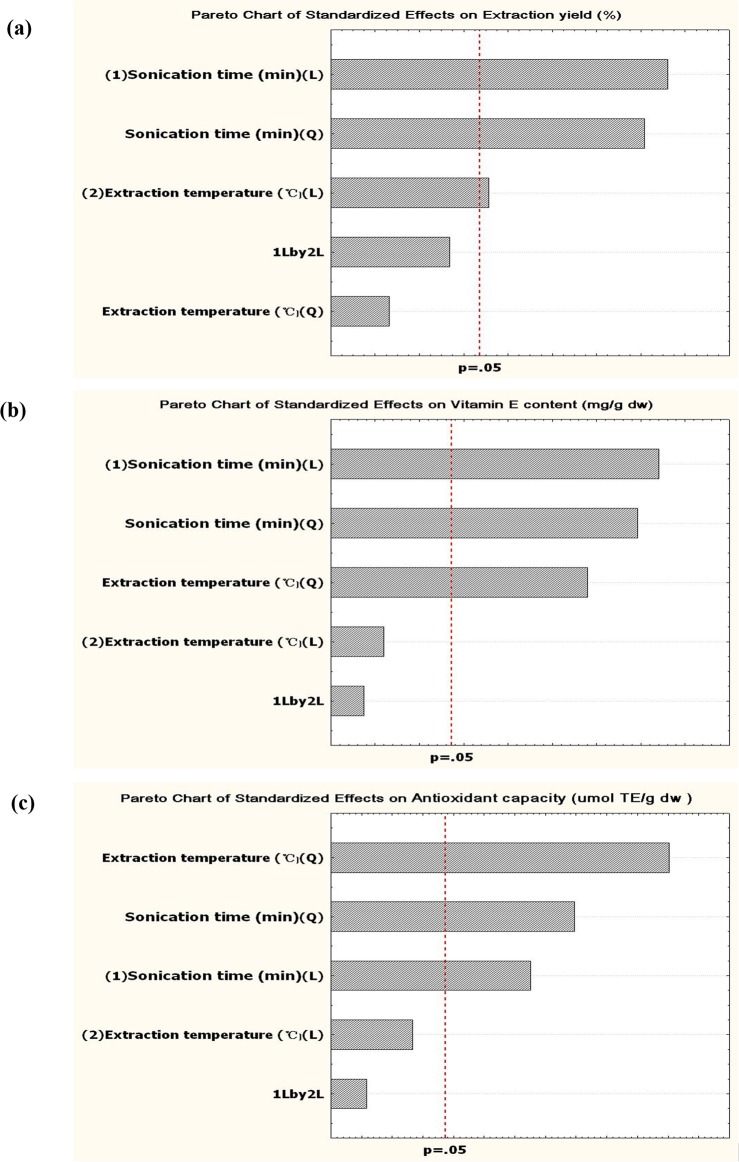
Pareto charts of standardized effects show significant variables on (a) extraction yield, (b) vitamin E, and (c) antioxidant capacity of raspberry seed oil obtained using ultrasonic-assisted extraction. The vertical dashed line indicated the level of significance at p = 0.05. L: linear effect; Q:quadratic effect.

Extraction yields of raspberry seed oil obtained under different UAE conditions are listed in Table 1. The lowest extraction yield of 17.77% was detected in test 5 under UAE conditions of 50°C for 10 min, which was the shortest extraction time in our test. The highest extraction yield of 23.01% was observed in test 9 under UAE conditions of 50°C for 30 min. Regression analysis and ANOVA results showed that the R square for extraction yield was 93% and lack of fit was insignificant with value of 0.21, indicating that the fitted model for the extraction yield of raspberry seed oil subjected to UAE is both adequate and reliable. Pareto charts were used to identify the most significant variables, as shown in Fig 1. The vertical dashed line represents the significance level at *p* = 0.05, and variables with a bar length beyond the dotted line are significant. The pareto chart in Fig 2(A) suggests that sonication time and extraction temperature are both critical variables in the UAE process, along with the quadratic effect of sonication time (*p*<0.05). The fitted second order polynomial model is listed below:

$$Y_{Extraction\;yield}=22.49+{0.99*X}_{1}+{0.39*X}_{2}-{0.66*X}_{1}^{2}-{0.44*X}_{12}-{0.04*X}_{2}^{2}$$

**Fig 3:**
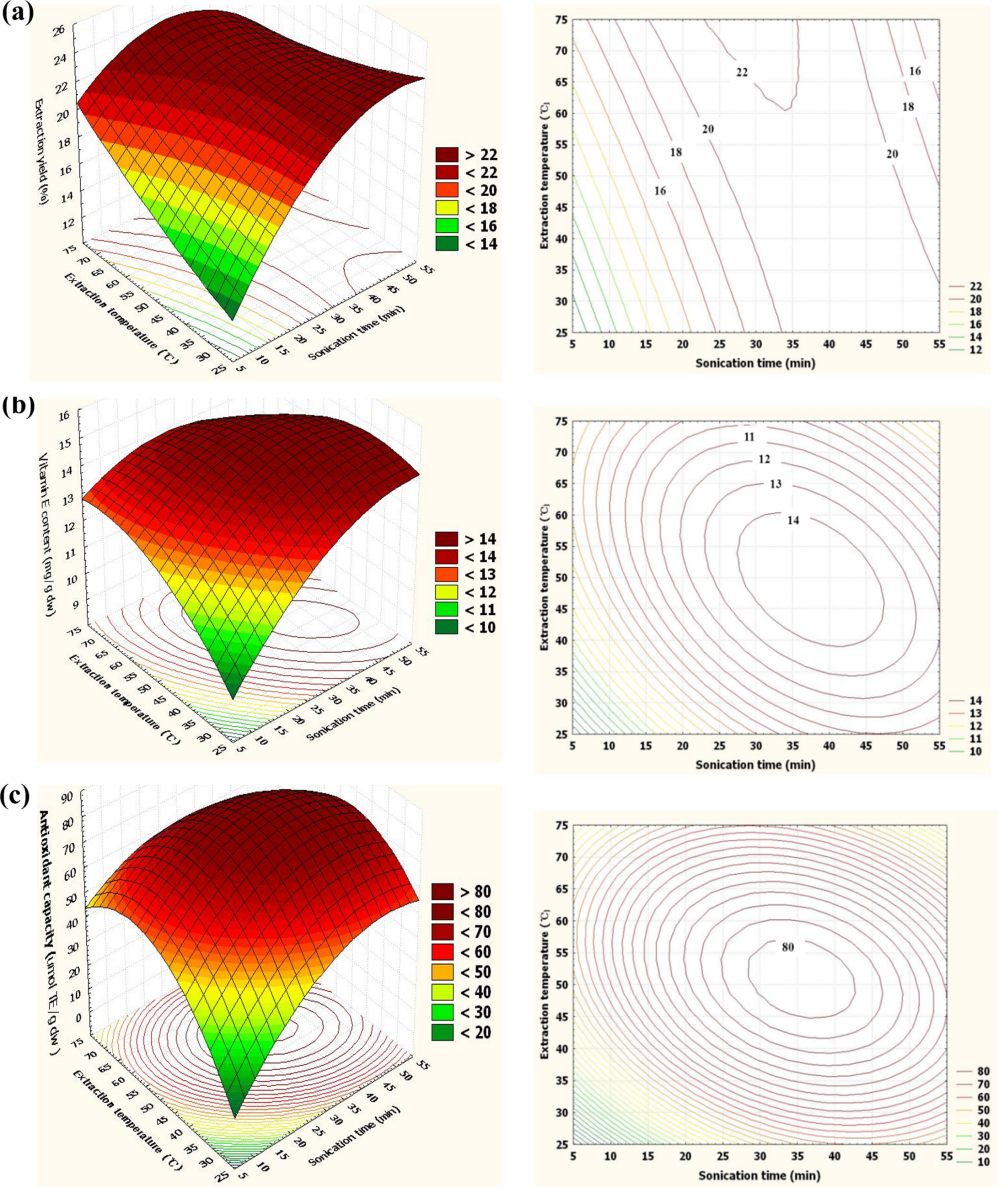
Three-dimensional surface and contour plots reflect the relationship of process variables on (a) extraction yield, (b) vitamin E content, and (c) antioxidant capacity of raspberry seed oil under ultrasonic-assisted extraction.

Where *X*_*1*_ is sonication time and *X*_*2*_ is extraction temperature. The three-dimensional surface plot showing the extraction yield of raspberry seeds subjected to UAE is shown in Fig 3(A). The saddle presented in Fig 3(A) indicates that the optimal conditions for UAE of raspberry seeds followed a rigid line instead of falling within a specific range [18]. According to the contour plot shown in Fig 3(A), sonication time showed a more ascend slope and combined with the parato chart as shown in Fig 3(A), confirming a more significant effect on extraction yield of raspberry seeds, and a higher extraction temperature reduced the sonication time required to achieve a satisfactory extraction yield. For example, based on the contour plot in Fig 3(A), an extraction yield of 20% was obtained at a low extraction temperature of 25°C for 35 min or at a high temperature of 70°C for 18 min.

Compared to conventional methods such as Soxhlet extraction (SE), UAE is more convenient as well as faster. Ultrasound generates and passes energy toward a material while simultaneously inducing an acceleration movement. This results in a cavitation effect that can damage to plant cell walls and let compounds through. Beside ultrasonic energy, heat can increase the speed of the extraction process. As confirmed in our results, extraction yield was substantially increased at high temperature with reduced sonication time, and a similar conclusion was also offered by Stanisavljevic et al. [23].

### Effects of process variables on vitamin E content of raspberry seed oil subjected to UAE

Vitamin E contents obtained under various UAE conditions are presented in Table 1. Regression coefficients as well as analysis of variance (ANOVA) for the fitted model of vitamin E content are calculated, and R square was 0.89 and lack of fit was insignificant with values of 0.14, confirming good fitness of the obtained data to the second order model. The pareto chart showing the standardized effects of the process variables for vitamin E content under UAE conditions is presented in Fig 2(B). The linear effects of sonication time on vitamin E content extraction as well as the quadratic effects of sonication time and extraction temperature were the most significant. The fitted model for the vitamin E content of raspberry seed oil subjected to UAE is listed below:

$$Y_{VE\;content}=14.84+{0.40*X}_{1}+{0.14*X}_{2}-{0.27*X}_{1}^{2}-{0.20*X}_{12}-{0.24*X}_{2}^{2}$$

Where *X*_*1*_ is sonication time and *X*_*2*_ is extraction temperature. Response surface plot for vitamin E content is presented in Fig 3(B). Vitamin E content increased as the extraction temperature and sonication time increased. Vitamin E content greater than 14 mg/g was generally obtained within the sonication time range of 27.5–46 min and extraction temperature range of 40–60°C. According to the GC-MS results, *α*- and *γ-*tocopherols were the primary forms of vitamin E contained in raspberry seeds. Zigoneanu et al. [24] obtained stable *α-* and γ-tocophrols from bran oil using MAE at 100°C, but our research found that vitamin E content decomposed after sonication treatment for longer than 46 min.

### Effects of process variables on antioxidant capacity

The antioxidant capacities of raspberry seed oil under different UAE conditions are presented in Table 1. Raspberry seed oils showed good antioxidant capacity, which ranged from 60.11% to 85.38% according to UAE conditions. The ANOVA results showed that model significance, R square value for antioxidant capacity were 0.90 and 0.0022, respectively, and lack of fit was 0.14, indicating that the fitted model for antioxidant capacity of raspberry seed oil subjected to UAE is highly reliable. The pareto chart for antioxidant capacity of raspberry seed oil subjected to UAE is shown in Fig 2(C), and the quadratic effects of both extraction temperature and sonication time as well as the linear effect of sonication time were highly significant. The fitted model for antioxidant capacity of raspberry seed oil subjected to UAE is listed below:

$${\text{Y}}_{\text{AC}}=80.50+{3.78*\text{X}}_{1}-{1.84*\text{X}}_{2}-{3.25*\text{X}}_{1}^{2}+{1.88*\text{X}}_{12}-{4.38*\text{X}}_{2}^{2}$$

Where X_1_ is sonication time and X_2_ is extraction temperature. Response surface plot for antioxidant capacity is shown in Fig 3(C). A good quadratic effect is shown in Fig 3(C), and antioxidant capacity improved with increased extraction temperature and sonication time. Maximal antioxidant capacity was generally attained when the extraction temperature and sonication time approached 50°C and 35 min, respectively, and it slightly decreased when the extraction temperature or sonication time was reduced. Vitamin E was considered as the main antioxidant compound in raspberry seed oil, whereas other compounds such as anthocyanins and flavonoids present in ethanol extract of raspberry seed oil highly contribute to antioxidant capacity [25]. The antioxidant capacity of raspberry seed oil may have been reduced due to the decomposition of thermal-sensitive polyphenol compounds.

### Optimization of UAE process, verification, and comparative experiment

**Fig 4:**
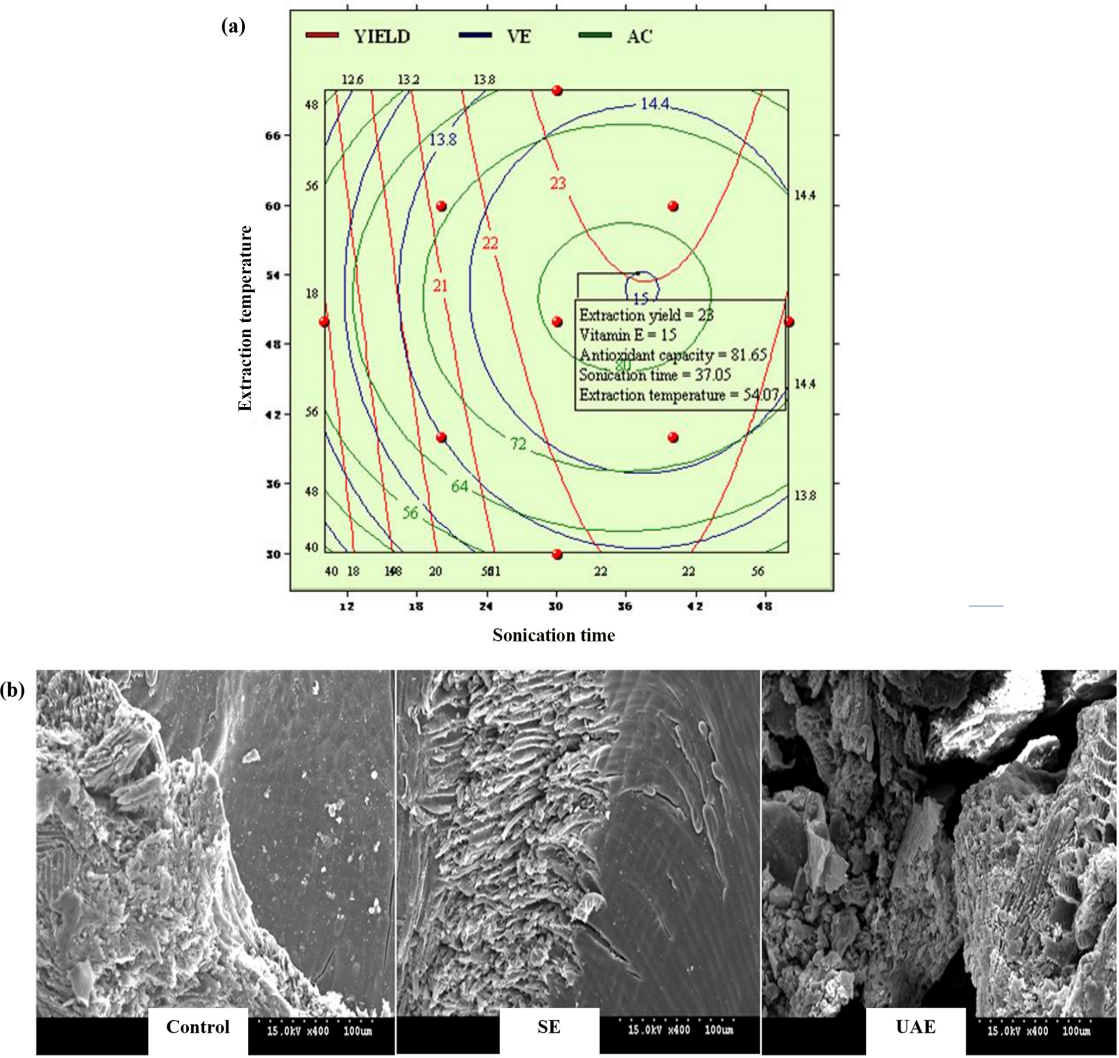
(a) Overlapped contour plot for the optimization of ultrasonic-assisted extraction (UAE) of raspberry seed. (b) Scanning electron microscopy images of raspberry seed tissues before and after ultrasonic-assisted extraction (UAE) and Soxhlet extraction (SE) processing.

According to canonical analysis, stationary points for vitamin E content and antioxidant capacity of raspberry seed oil subjected to UAE were maximal points, whereas the extraction yield was a saddle point. Overlapped contour plot (Fig 4A) was employed for the optimization of extraction yield, vitamin E content, and antioxidant capacity simultaneously. Obtained optimal UAE conditions were a sonication time of 37 min and an extraction temperature of 54°C, and the predicted maximal values for extraction yield, vitamin E content, and antioxidant capacity were 23%, 15 mg/g dw, and 81.65 μmol TE/g dw, respectively. Verification experiment was carried out under the predicted optimal conditions, and experimental values of extraction yield (22.78 ± 0.27%), vitamin E content (15.21 ± 0.59 mg/g dw), and antioxidant capacity (80.94 ± 1.83 μmol TE/g dw) were obtained. The predicted and experimental values were not significantly different, confirming that our prediction model is adequate and reliable while the application of RSM to UAE of raspberry seed oil was successful.

Cold press is one of the most commonly used methods for raspberry seed extraction at present [11,21,25]. This traditional method is capable of preserving substantial amounts of antioxidants, but it is complicated and has a low yield output (less than 5%). In the present study, SE was employed as a comparative experiment for raspberry seed separation. After heating raspberry seeds at 80°C for 4 h using a Soxhlet apparatus, an extraction yield of 18.15 ± 0.33%, vitamin E content of 13.48 ± 0.47 mg/g dw, and antioxidant capacity of 55.05 ± 2.46 μmol TE/g dw were obtained. Compared to our highly efficient UAE method, SE required that the boiling point of the solvent be maintained for a longer time period, resulting in decomposition of thermal-sensitive compounds [12].

### Morphological comparison of raspberry seed tissues after UAE and SE

Fig 4(B) shows a set of SEM images of raspberry seeds at a magnification factor of 400 after UAE and SE. Raspberry seed tissues subjected to SE showed intact and compact structures as well as no destruction of cell walls as compared to untreated samples (control). On the other hand, the microstructure of raspberry seed tissues after UAE was greatly disintegrated. Specifically, raspberry seed tissues became porous, and some micro-fractures and hollow openings were generated by UAE. Vinatoru [26] explained that ultrasonic waves can induce the formation and collapse of bubbles within very short time periods. In the vicinity of plant membranes, such a bubble collapse may result in strong shear forces that can cause micro-fractures in plant tissues.

### Fatty acid composition of raspberry seed oil

**Fig 5:**
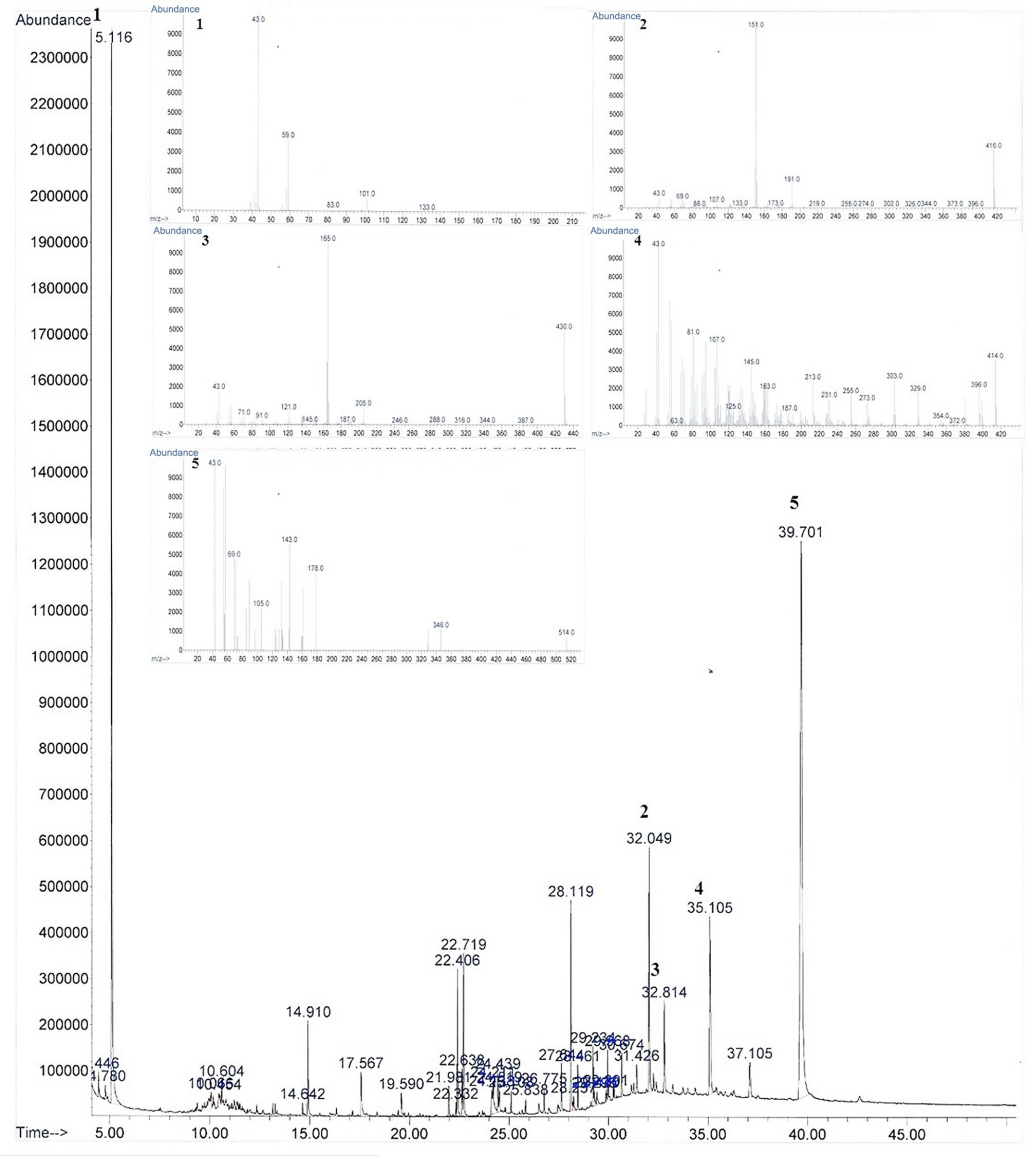
Gas chromatograph mass spectrometry (GC-MS) profile of essential oil from raspberry seed. Peak 1, Linoleic acid; Peak 2, γ-Linolenic acid; Peak 3, γ-Tocopherol; Peak 4, α-Tocopherol; Peak 5, γ-Sitosterol.

Fig 5 shows the GC-MS profile for raspberry seed oil. And Table 2 presents the fatty acid compositions of raspberry seed oils subjected to UAE at 54°C for 37 min as well as conventional SE at 80°C for 4 h. Fatty acid compositions of the extracts were highly similar, although oil subjected to UAE showed a higher polyunsaturated fatty acid composition as well as lower saturated fatty acid composition compared to oil subjected to SE (Table 2). Total saturated fatty acid content of raspberry seed oil subjected to UAE was 2.45%, whereas monounsaturated fatty acid (MUFC) and polyunsaturated fatty acid (PUFA) contents were 0.55% and 92.25%, respectively. Linoleic acid and γ-linolenic acid (GLA) were the dominant fatty acids in raspberry seed oil, constituting greater than 59% and 33% of the total fatty acid content, respectively. ω-6 fatty acids, especially linoleic acid, are the main fundamental material involved in the synthesis of structural lipids as well as cellular membranes, and various studies [27–29] have reported that consumption of GLA may be a new strategy for the treatment and prevention of certain chronic diseases such as cardiovascular disease, cancer, and inflammatory diseases. However, edible GLA is rarely found in natural foods, and significant commercial sources of GLA at present include oil of borage (20–26%), black current (15–18%), and evening primrose (8–12 GLA) as reported by Kapoor and Huang (2006). The present study confirmed a substantially high GLA content in raspberry seeds, suggesting that raspberry seed is a good source of GLA. In addition, GLA from raspberry seeds is natural and edible, which increases the applicability of raspberry seeds as a functional food and additive for cosmetics.

**Table 2:** Fatty acid compositions of ultrasonic-assisted extracted oil (UAE) and soxhlet extracted (SE) raspberry seed oil.

No.		Fatty acids	UAE (%)	SE (%)
1	C12:0	Lauric acid	0.02	0.02
2	C14:0	Myristic acid	0.06	0.05
3	C16:0	Palmitic acid	0.07	0.06
4	C18:0	Stearic acid	1.34	5.15
5	C20:0	Arachidic acid	0.59	0.50
6	C21:0	Heneicosylic acid	0.04	0.03
7	C22:0	Behenic acid	0.25	0.22
8	C23:0	Tricosanoic acid	0.03	n.d.
9	C24:0	Lignoceric acid	0.06	0.04
10	C17:1	Heptadecenoic acid	0.08	0.08
11	C18:2 _n = 6_	Linoleic acid	59.12	57.94
12	C18:3 _n = 3_	Linolenic acid	32.78	30.37
13	C19:1	Nonadecenoic acid	0.06	0.03
14	C20:1	Paullinic acid	0.41	0.38
15	C20:2	Eicosadienoic acid	0.35	0.27
Saturated fatty acid			2.45	6.07
Monounsaturated fatty acid			0.55	0.49
Polyunsaturated fatty acid			92.25	88.58
**Total**			96.14	95.14

n.d.: not detected

### Antioxidant activity

**Fig 6:**
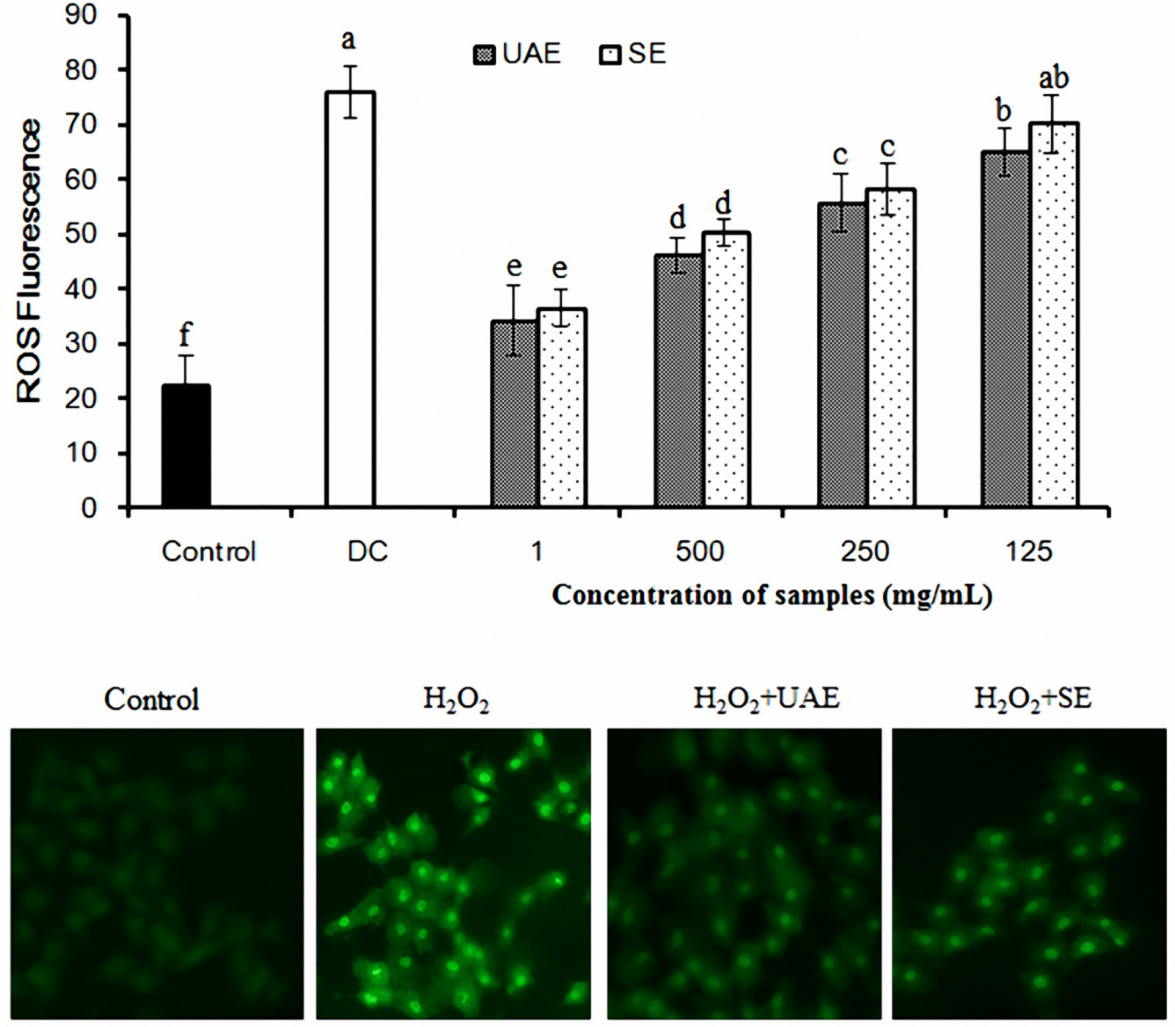
Inhibitory effect of extract from raspberry seed on H2O2-induced production of intracellular ROS of UAE and SE. RAW 264.7 macrophages were incubated in the presence or absence of H2O2. (A) The ROS levels in the macrophages were determined using fluorescence plate reader. The y axis of the ROS fluorescence represented the intensity of the fluorescent DCF in cell samples relative to unstained cells. The results are the means of three independent experiments. Bars with different alphabets are significantly different (P < 0.05). DC: H2O2-damaged group. (B) The ROS levels were monitored with laser scanning confocal microscope.

The free radical-scavenging activity of UAE was significantly higher than that of intact SE at any tested concentration (*P* < 0.05). The highest scavenging capacity of UAE at the concentration of 2.0 mg/mL for DPPH, ABTS, and NO with 31.4%, 59.5% and 75.3%, respectively (data not shown). The effect of UAE and SE on cellular ROS level in H_2_O_2_-stressed RAW 264.7 macrophages was assessed. As shown in Fig 6A, the production of intracellular ROS was indirectly measured using non-fluorescent dichloro-dihydro-fluorescein diacetate (DCFH-DA), which was converted to fluorescent dichlorofluorescein (DCF) in the presence of ROS. Quantitative analysis of fluorescence intensity revealed that the intracellular ROS levels were significantly increased by H_2_O_2_ treatment (*P* < 0.05). UAE pretreatment significantly reduced ROS generation induced by H_2_O_2_ in a dose dependent manner compared with that in the H_2_O_2_-damaged group (*P* < 0.05). For the intracellular ROS localization, RAW 264.7 macrophages was observed at 630× magnification under oil immersion with the laser scanning confocal microscope. As shown in Fig 6B, the control cells displayed weak green fluorescence, whereas the cells treated with H_2_O_2_ showed striking green fluorescence, reflecting the increase of intracellular ROS levels. In contrast, this elevation was almost reversed by UAE and SE (2 mg/mL) pretreatment. These results suggested that extract of raspberry seed was a potential free radical scavenger.

## Conclusion

Response surface methodology was successfully applied to the UAE of raspberry seed oil for the simulation and administration of extraction, improvement of oil recovery, and reduction of unnecessary nutrient loss. The ANOVA results show that sonication time was the most significant factor affecting raspberry seed oil extraction. Optimal conditions include a sonication time of 37 min and extraction temperature of 54°C. Different extraction methods had significant effects on raspberry seed oil quality. Specifically, UAE was able to provide a higher content of beneficial unsaturated fatty acids (UFA), whereas conventional Soxhlet extraction (SE) resulted in a higher amount of saturated fatty acids. Moreover, raspberry seed oil contained abundant amounts of edible linoleic acid and linolenic acid, which shows it may be a valuable edible source of natural GLA products. This work also demonstrated that UAE could attenuate H_2_O_2_-induced oxidative stress injury in macrophages. The protective effects of UAE partly depended on the combination of alleviating intracellular ROS production and restoring the activities of endogenous antioxidants.

## Supporting Information

S1 Fig DPPH-scavenging capacity (A), ABTS scavenging capacity (B), NO scavenging activity (C) of UAE and SE. All measurements are expressed as means ± SD of three separate determinations.
(DOCX)
