# Supplementary material for: Ultrasonic-Assisted Extraction of Raspberry Seed Oil and Evaluation of Its Physicochemical Properties, Fatty Acid Compositions and Antioxidant Activities
Source: PLoS One. 2016 Apr 27;11(4):e0153457. doi: 10.1371/journal.pone.0153457 (PMC4847764; doi:10.1371/journal.pone.0153457)
Supplement: S1 Fig — DPPH-scavenging capacity (A), ABTS scavenging capacity (B), NO scavenging activity (C) of UAE and SE. All measurements are expressed as means ± SD of three separate determinations. (DOCX) [file pone.0153457.s001.docx]

**Supplementary material**

**
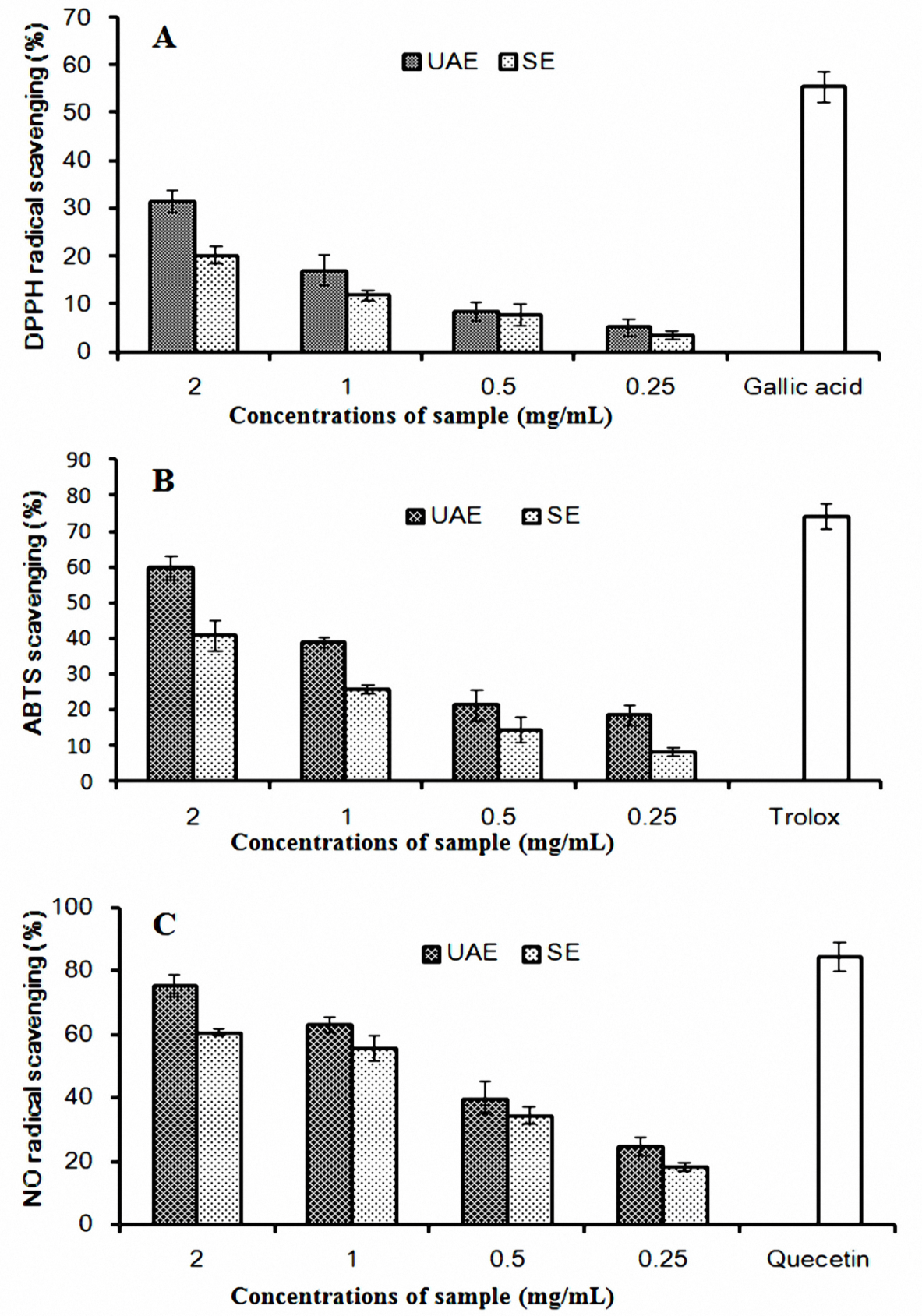
**

DPPH-scavenging capacity (A), ABTS scavenging capacity (B), NO scavenging activity (C) of UAE and SE. All measurements are expressed as means ± SD of three separate determinations.
